# Examining EFL vocational school teacher resilience in the Chinese context: a structural equation modeling approach

**DOI:** 10.3389/fpsyg.2025.1444979

**Published:** 2025-11-19

**Authors:** Siyu Duan, Jianli Cui, Xiaoxue Li, Fangliang Wang, Miaoyue Xia

**Affiliations:** 1 College of Foreign Languages, Jilin Agricultural University, Changchun, China; 2 School of Foreign Languages, Changchun Guanghua University, Changchun, China; 3 School of Foreign Languages, Soochow University, Suzhou, China; 4 School of General Education and Practice, Wuhan Technical College of Communications, Wuhan, China; 5 School of Foreign Studies, South China Agricultural University, Guangzhou, China

**Keywords:** resilience, EFL teacher, vocational school, EFL Teacher Resilience Scale, structural equation modeling approach

## Abstract

Teacher resilience has recently received significant attention in psychology and general education. However, the research on English as a foreign language (EFL) teacher resilience, particularly among vocational school teachers, has been understudied. There is still a lack of exploration regarding the internal structure of EFL vocational school teacher resilience in the Chinese context. To address this gap, this study employed a structural equation modeling approach to investigate the structure of resilience among EFL vocational school teachers. The EFL Teacher Resilience Scale was completed by 265 Chinese EFL vocational school teachers. Confirmatory factor analysis revealed a tri-dimensional structure of EFL vocational school teacher resilience involving professional, emotional and social and cultural dimensions. Additionally, the findings of this research indicated a significantly high level of EFL vocational school teacher resilience, both overall and across its three dimensions. These findings provided implications for the sustainable professional development of EFL vocational school teachers and suggestions for further research in this field.

## 1 Introduction

Teaching has long been acknowledged as a highly stressful field across numerous countries from everlasting (Boldrini et al., 2018; Chen et al., 2023; Liu and Chu, 2024; MacIntyre et al., 2020; Mercer, 2023). This stress is notably exacerbated in the specialized context of English as a Foreign Language (EFL) teaching within vocational schools. Beyond the burdens of a demanding workload and rigors of promotion for college teachers (Duan et al., 2023; Yu et al., 2022), EFL vocational school teachers also contend with a distinct set of professional-specific pressures. Vocational education, as an integral component of higher education, has reached a flourishing stage in its development and plays an equally significant role in general higher education (Chen et al., 2023; Özdemir et al., 2024; Wen and Zhang, 2021), contributing to the promotion of educational equity (Ye et al., 2023). The practical and application orientation of vocational education necessitates specific requirements for EFL teachers, reflecting the growing expectations from social, institutions and students. Vocational EFL teachers are expected to embody a “tri-professionalism” attribute, which entails a nuanced command of English language and cultural knowledge, a comprehensive understanding of occupational pedagogy knowledge, and an extensive grasp of the domain-specific knowledge they teach (Wang, 2016). In addition, EFL teachers are required to seamlessly integrate English for General Purposes with English for Occupational Purposes, thereby fostering an enhanced level of students' engagement and motivation in their learning endeavors. However, insufficient attention has been given to English curricula at both the institutional and student levels, as vocational schools are application-oriented and primarily focus on cultivating technical and practical expertise and occupational skills (Ye et al., 2023; Warwas and Helm, 2018). Consequently, English teachers frequently find themselves in a state of “marginalized.” Given that vocational schools are frequently perceived as a fallback option for students, the confluence of suboptimal English proficiency, diminished learning motivation, and disciplinary issues among the students has presented formidable obstacles to the pedagogical effectiveness of EFL vocational school teachers. The EFL vocational school teacher resilience in that context is a crucial ability for effectively utilizing diverse resources to cope with stress, maintain teaching enthusiasm, enhance teacher retention, and promote sustainable professional development (Barnová et al., 2023; Day and Gu, 2009; Liu et al., 2024; Tait, 2008). However, EFL vocational school teacher resilience has not garnered the attention it merits. To bridge the research gap, this study built on previous definitions and posited that EFL vocational school teacher resilience was a quality/ability enabling teachers to recover from stress and challenges and a process through which teachers interacted with their surrounding environment (Duan et al., 2023; Liu et al., 2024; Liu and Chu, 2022a). Furthermore, this study examined the internal structure and the level of resilience among EFL vocational school teachers to formulate pertinent recommendations for bolstering their overall wellbeing and sustaining their professional development.

## 2 Literature review

### 2.1 Conceptualizing language teacher resilience

Research on EFL teacher resilience has gradually gained prominence, and existing review offers valuable insights into the overall landscape of this field (Liu and Chu, 2022b). Given the rising prevalence of research on teacher resilience in the recent two decades, there is a crucial imperative to conceptualize language teacher resilience (Chen, 2024; Duan et al., 2023; Liu et al., 2024; Liu and Chu, 2022a). Despite the lack of consensus regarding the definition, existing literature has identified a shared agreement that a combination of distinct attributes can characterize language teacher resilience. First, language teacher resilience is a crucial concept that refers to the intrinsic capacity or quality that empowers teachers to navigate the challenges and adversities within their professional field. The characteristic feature lies in its capacity to effectively manage and mitigate the impact of stress, rebound from setbacks and hardships, restore equilibrium, and achieve success (Gu and Day, 2013; Brunetti, 2006; Day and Hong, 2016; Li and Lv, 2022). Second, it is crucial to acknowledge that language teacher resilience is context-specific (Gao et al., 2022; Gu and Li, 2013; Liu et al., 2024). Recognizing teacher resilience as a contextual attribute highlights the significance of considering the specific social, cultural, institutional, and pedagogical factors that shape teacher resilience (Gu, 2014; Kärner et al., 2021b). It requires a deeper understanding of the specific structure in different teacher groups and an examination of how different contexts may promote or hinder the development of teacher resilience. The social beliefs and expectations and important others (such as leaders, parents, colleagues and students) have been evidently proven as the contextual sustenance of teacher resilience (Beltman et al., 2011; Day and Gu, 2007; Duan et al., 2023). Third, language teacher resilience is a dynamic process shaped by a complex interplay and interactions with their environment (Sammons et al., 2007). The resilience of language teachers is shaped by the ongoing interconnection between their internal features, such as self-efficacy (Wang et al., 2024; Wang and Pan, 2023) and emotions (Gan et al., 2022), as well as the diverse educational ecosystems in which they live and work. The dynamic process of teacher resilience is characterized by temporal changes within individual teachers (Duan et al., 2023) and variations across different years of teaching experience (Day and Gu, 2007). Consequently, the EFL vocational school teacher resilience can be understood as a multifaced quality that enables teachers to overcome challenges in English teaching at vocational schools effectively. This resilience is also a process shaped by their interplay with the elements of the working environment and subject matter expertise while fostering their continuous commitment to professional learning and growth amidst these difficulties.

### 2.2 The studies on teacher resilience in vocational schools

The exploration of vocational school teacher resilience is crucial, as it has the potential to enhance their abilities to address challenges, including dealing with setbacks from poor-performing students, little control over the occupational environment, insufficient teaching resources, low social recognition, and negative leadership (Barnová et al., 2023; Boldrini et al., 2018; Chen et al., 2023). Despite the recognized significance of this area, research on vocational school teacher resilience has been notably limited. The limited existing research has commenced exploring the influential factors (Barnová et al., 2023; Boldrini et al., 2018; Kärner et al., 2021b) and strategies to enhance resilience (Sappa and Barabasch, 2020), offering in-depth viewpoints for this study.

Given the increasing recognition of the influential role of individual and environmental factors on vocational school teacher resilience, researchers have begun to examine the resources and challenges affecting vocational school teacher resilience within the complex vocational education ecosystem (Barnová et al., 2023; Boldrini et al., 2018; Kärner et al., 2021b). In this regard, Barnová et al. (2023) employed the Connor-Davidson Resilience Scale (CD-RISC-25^SLOVAK^) to assess the resilience of 474 vocational school teachers in Slovakia. They aimed to delineate the relationship between teacher resilience and years of teaching experience. The findings underscored the pivotal role that years of teaching experience exerted on the construct of teacher resilience, revealing that teachers with more years of teaching experience exhibited higher levels of resilience than novice teachers. The effect was particularly evident across five dimensions: Hardiness, Coping, Adaptability/Flexibility, Meaningfulness/Purpose, and Optimism. Boldrini et al. (2018) conducted a qualitative approach, engaging in interviews with 37 vocational education and training teachers from a socio-ecological perspective. This study specifically sought to identify the adverse conditions and situations and the resources and protective factors that influence teacher resilience within their working environment. The findings revealed that teachers perceived various difficulties, including context-related challenges (e.g., low social recognition, conflicts with colleagues and challenging students), individual challenges (e.g., excessive emotional involvement and fear of being judged incompetent) and teaching professional challenges (e.g., instructional challenges and teaching role challenges). Teachers also identified several resources, including context-related resources (e.g., opportunities for training and positive school leadership and support) and individual resources (e.g., flexibility, subject-specific teaching skills and intrinsic motivation and vocation). Kärner et al. (2021b) embarked on a study from a social perspective in Germany involving 307 trainees and qualified teachers from both vocational and general schools to explore the contextual resources that contributed to teacher resilience during their training. The findings underscored that mentors, partners, fellow trainees, and colleagues emerged as primary sources of social support. In summary, these insights have profound implications for understanding the EFL vocational school teacher resilience level.

A variety of strategies and recommendations in the literature have been proposed for policymakers and school administrators to provide social support for enhancing vocational school teacher resilience (Barnová et al., 2023; Sappa et al., 2019). It is worth noting that empirical studies have explored interventions to build vocational school teacher resilience. A case in point is the research conducted by Sappa and Barabasch (2020), who employed a mixed-methods approach to examine the efficacy of a forum-theater workshop on building resilience among 230 in-service vocational school teachers in Canton of Ticino. The results highlighted that implementing such a forum-theater workshop held significant potential for enhancing creativity and resilience among teachers.

### 2.3 Understanding the structure of teacher resilience

It is clear from the extensive evidence that teacher resilience is a multidimensional construct (Chen, 2024; Chu and Liu, 2022; Liu et al., 2024; Mansfield and Wosnitza, 2015); however, there exist different viewpoints regarding the specific internal structure of teacher resilience. This diversity contributes to a more nuanced and comprehensive understanding of teacher resilience, as it encourages the examination of diverse theoretical frameworks and research participants across various teaching settings (Kärner et al., 2021a; Li et al., 2019; Liu and Chu, 2022a, 2024). The related empirical evidence from several studies indicated a three-dimensional structure of teacher resilience. An obvious example was the research of Li et al. (2019), which employed a mixed-methods approach to examine teacher resilience among 455 primary and secondary school teachers in Beijing, China. The findings unveiled that the resilience of Chinese teachers encompassed three dimensions, namely commitment and motivation for teaching, sense of self-efficacy, and job fulfillment. The study of occupational teacher resilience has also revealed that occupational teacher resilience, specific to resilience competencies, consists of three dimensions (Kärner et al., 2021a). Kärner et al. (2021a) contributed to this understanding by investigating three fundamental dimensions from the survey outcomes of 131 vocational trainees and qualified teachers in Germany. Three resilience competencies were dynamism, flexibility, and resistance. In the field of language teacher resilience, Liu and Chu (2022a) employed Connor-Davidson resilience scale (Connor and Davidson, 2003) to adapt a questionnaire for measuring Chinese EFL teacher resilience. Their study focused on a sample of 658 Chinese senior high school EFL teachers, with the aim to unveil the structure of resilience within this particular demographic. Through exploratory and confirmatory factor analysis, it was revealed that teacher resilience comprised three dimensions: tenacity, optimism, and coping style. Additionally, Liu and Chu (2024) utilized the Multidimensional Teachers' Resilience Scale (Mansfield and Wosnitza, 2015) to examine the structure of EFL teacher resilience in China, with a sample of 539 junior high school EFL teachers. Through their analysis, three distinct factors emerged that were named as grit, professional competence, and sociability.

Additionally, evidence from a spectrum of studies posited that teacher resilience was structured around four core dimensions. Daniilidou and Platsidou (2018) conducted a study with 136 secondary education teachers and 146 primary school teachers in Greece, employing both the Connor-Davidson Resilience Scale (Connor and Davidson, 2003) and the Resilience Scale for Adults (Friborg et al., 2005) to examine the resilience. Their findings showed that teacher resilience comprised personal competencies and persistence, spiritual influences, family cohesion, as well as social skills and peer support. In another series of studies, Mansfield et al. (2012) collected data through both paper and online version of the survey, posing the question “*How would you describe a resilient teacher?”* to 200 graduating and early career teachers. Their research revealed a four-dimensional teacher resilience encompassing emotional, professional, motivational, and social aspects of resilience. Mansfield and Wosnitza (2015) developed the Multidimensional Teachers' Resilience Scale to examine the internal structure of teacher resilience. The scale consisted of 26 items distributed across four dimensions: motivation, social, emotional and professional dimensions. This scale has received recognition and has been widely adopted by researchers in this field. Peixoto et al. (2020) and Chen and Lee (2022), for example, have employed this scale to examine further and validate the four-dimensional structure of teacher resilience. In the context of language teacher resilience, Liu et al. (2024) adapted the Multidimensional Teachers' Resilience Scale to create a contextually relevant resilience scale for Chinese language teachers. Their research, using a sample of 3,992 high school EFL teachers in China, revealed that EFL teacher resilience can be categorized into four dimensions: professional, emotional, social, and cultural dimensions. The cultural dimension, which was newly developed, reflected the unique context and role of language teacher resilience.

Moreover, the results of several studies indicated that teacher resilience could be conceptualized as a multidimensional construct comprising five dimensions. Chen (2024), for instance, developed a teacher resilience inventory using a sample of primary school teachers. The findings revealed that teacher resilience encompassed five dimensions: physical resilience, emotional resilience, psychological resilience, social resilience and spiritual resilience.

In summary, the quest to delineate the internal structure of teacher resilience has been a subject of considerable scholarly endeavor. These efforts are driven by the recognition that understanding the components of teacher resilience is instrumental in discerning the distinctive attributes across various educational stages and disciplines. Such insights are invaluable for devising targeted intervention strategies to nurture and enhance teacher resilience from multiple dimensions. The exploration of teacher resilience has spanned diverse teacher groups, including those in primary and secondary schools, vocational schools and language teaching contexts. These studies have provided a foundation for understanding the nuances of resilience in different educational contexts. However, there is still ample opportunity for further exploration within specific teacher groups, particularly at different certain stages and disciplines. Therefore, that motivates the need to focus on EFL vocational school teachers to examine the internal structure of their resilience, to identify specific dimensions for measuring the level of teacher resilience, to uncover the characteristics unique to this group, and to propose strategies for enhancing their resilience. In light of both theoretical and practical research objectives, the current study presented the following two research questions:

1. What is the structure of EFL vocational school teacher resilience?
2. What are the overall and dimensional profiles of EFL vocational school teacher resilience?

## 3 Methods

### 3.1 Research participants

The valid sample for this study was comprised of 265 EFL teachers from vocational schools, who were invited to participate in this survey through the convenience sampling method (Taherdoost, 2016). The participants were from various regions in China, including the provinces of Jiangsu, Liaoning, Henan, Jilin, Shandong, Zhejiang, Hainan, and Guangdong; the Xinjiang Uygur Autonomous Region; and the municipalities of Beijing and Shanghai. The gender distribution within the sample was predominantly female, with 222 female participants and 43 male participants. In terms of age distribution, the sample included 13 (4.9%) teachers aged 30 years or younger, 61 (23.0%) aged between 31 and 40 years, 146 (55.1%) aged between 41 and 50 years, and 45 (17.0%) aged over 50 years. The sample also represented a range of teaching experience levels: 19 (7.2%) teachers had 5 years of experience or less, 22 (8.3%) had 6–10 years of experience, 44 (16.6%) had 11–15 years, 71 (26.8%) had 16–20 years, and 109 (41.1%) possessed more than 20 years of teaching experience. In China, the vocational education system is divided into several levels, including secondary vocational schools (such as vocational high schools and technical schools) and higher vocational schools. The research participants in this study were from higher vocational schools; we used vocational schools for convenience.

### 3.2 Research instrument

As part of a series of studies, the EFL vocational school teacher resilience measure in this study was designed based on the *Language Teacher Resilience Scale* (Liu et al., 2024, 2025). The adapted scale encompassed four dimensions: professional dimension (*n* = 5), emotional dimension (*n* = 5), social dimension (*n* = 5), and cultural dimension (*n* = 5). Given that the original scale was developed and tailored for the Chinese educational setting, we built upon it for our research. However, to further enhance its applicability to the specific teaching environment of vocational schools, we made some targeted adaptations. Certain items were rephrased to ensure relevance to the vocational school teaching context. For example, the item “In the face of the increasing expectations and requirements of the public for teachers, I can work hard and fulfill my mission” was reworded into “In the face of the increasing expectations and requirements imposed by the reform of English education in vocational schools, I can work hard and fulfill my mission.” The items were rated on a 6-point Likert scale varying between 1 (not true at all) and 6 (true all the time).

### 3.3 Data collection

The data were obtained in November 2023 via Wenjuanxing, an online survey platform. The ethics approval was waived since this study did not involve intervention and was low risk. Prior to collecting the data, the research participants were informed of the research objectives and ensured voluntary participation in the survey. They were also guaranteed that their responses would be handled with anonymity and confidentiality. The participants were invited to share the questionnaire link with their colleagues. Altogether, 294 participants provided their responses to the online questionnaire, the valid response rate being 90.1%.

### 3.4 Data analysis

The data were recorded and analyzed using SPSS 26.0 and AMOS 23.0 software. The outliers and incomplete responses were excluded in advance. After eliminating 29 outliers, a total of 265 complete and valid responses were retained in the final analysis.

Initially, a univariate normality test was conducted to check whether the data were normally distributed. An item analysis was then performed to ensure the discriminant validity of the items. Subsequently, confirmatory factor analysis (CFA) was carried out to investigate the underlying structure of the EFL vocational school teacher resilience. The model fit was evaluated following the guidelines proposed by Kline (2016) and Browne and Cudeck (1993): Comparative Fit Index (CFI) > 0.90, Tucker-Lewis Index (TLI) > 0.90, Root Mean Square Error of Approximation (RMSEA) < 0.10, and Standardized Root Mean Square Residual (SRMR) < 0.08. Descriptive analysis was then employed to explore the status quo of resilience among the participants.

## 4 Results

In this section, we presented the results on the resilience of EFL vocational school teachers. The results were divided into two main parts: the factorial structure of EFL vocational school teacher resilience and the levels of this resilience. These results provided valuable insights into the resilience profile of EFL teachers in the vocational school context.

### 4.1 The structure of EFL vocational school teacher resilience

#### 4.1.1 Normality and item analysis results

The analysis commenced with a test for univariate normality to assess the distribution of the collected data. The results, detailed in the Supplementary material, indicated that skewness values ranged from −1.723 to −0.757 and kurtosis values from −0.558 to 3.834, which fell within the benchmarks of |2.0| and |7.0|, respectively, suggesting a normal distribution of the data (Finney and DiStefano, 2006; West et al., 1995). Subsequently, item analysis was conducted to evaluate the discriminant validity of each item on the scale (Field, 2013). The top and bottom 27% of EFL teachers, based on their scores on the adapted 20-item EFL vocational school teacher resilience scale, were selected and compared using an independent samples *t*-test. The results of the independent samples *t*-test revealed significant differences between these two groups for each item (*p* < 0.001), confirming the appropriateness of all items for further analysis. Additionally, item-total correlation analysis was performed to examine the relationship between each item and the overall scale. The correlation coefficients were between 0.594 and 0.833, higher than the threshold of 0.30 (*p* < 0.001; Field, 2013). The detailed results of the independent samples *t*-test and item-total correlation analysis were available in the Supplementary material.

#### 4.1.2 Results of confirmatory factor analysis

**Figure 1:**
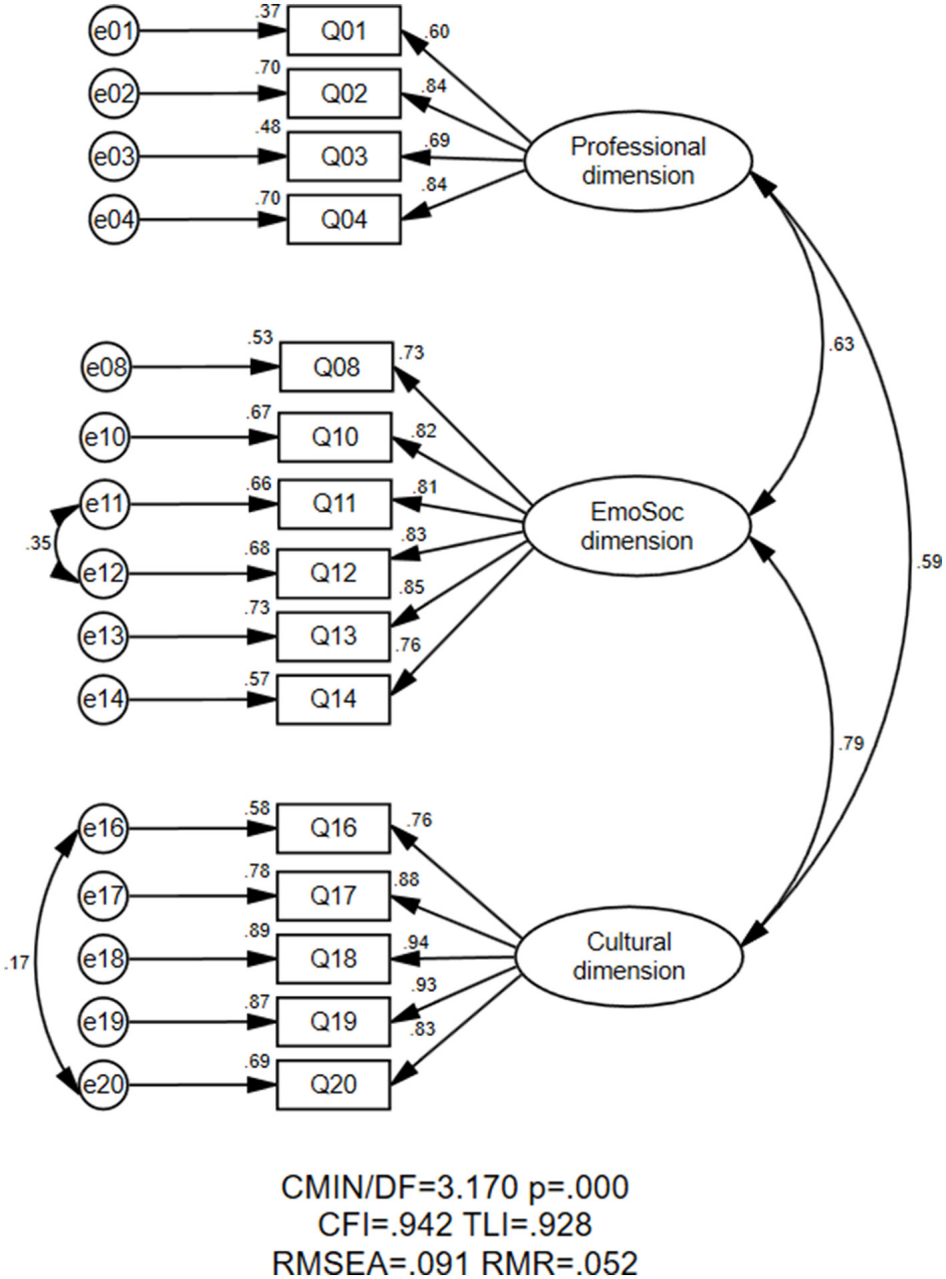
The final measurement model of EFL vocational school teacher resilience.

Confirmatory factor analysis (CFA) was conducted to evaluate the hypothesized four-factor structure of EFL vocational school teacher resilience. The initial CFA results did not yield satisfactory model fit indices for the proposed structure. Notably, the emotional and social dimensions showed extremely high intercorrelations (*r* = 0.97, *p* < 0.001), exceeding the square root of the average variance extracted (AVE) for both dimensions, an indication of unacceptable discriminant validity. Therefore, items of emotional dimension and social dimension were put together to form a new dimension. The results of CFA confirmed that the modified tri-factorial structure of EFL vocational school teacher resilience with 15 items displayed an ideal model fit (see Figure 1). Specifically, the RMSEA and SRMR were 0.091 and 0.068, respectively. The CFI and TLI both exceeded the threshold of 0.90. The results of CFA for the three subdimensions of EFL vocational school teacher resilience were available in Table 1.

**Table 1:** Results of CFA for the three subdimensions of EFL vocational school teacher resilience.

**Variables**	***χ^2^*/df**	**CFI**	**TLI**	**RMSEA**	**SRMR**
Professional dimension	0.452	1.000	1.008	0.000	0.008
Emotional and social dimension	3.190	0.983	0.969	0.091	0.024
Cultural dimension	1.537	0.998	0.996	0.045	0.008

**Figure 2:**
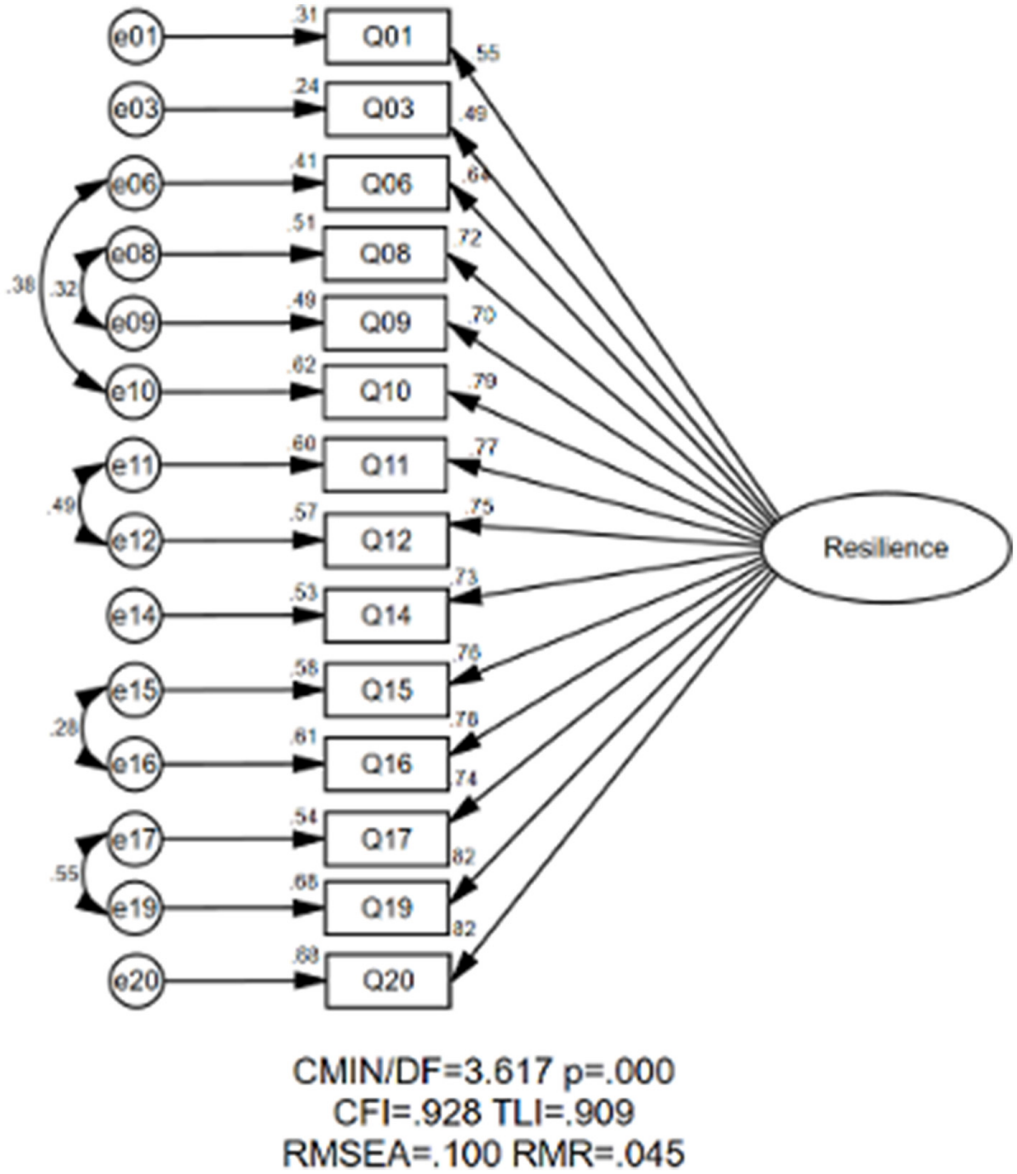
The one-factorial measurement model of EFL vocational school teacher resilience.

In addition, we also evaluated the one-factorial structure of EFL vocational school teacher resilience. The initial CFA results indicated unsatisfactory model fit indices for this structure. As Figure 2 illustrates, the modified one-factorial structure exhibited poorer model fit indices compared to the tri-factorial structure: RMSEA = 0.100, SRMR = 0.053, CFI = 0.928, TLI = 0.909. Considering both the theoretical framework and statistical analysis, the tri-factorial structure was chosen as the final measurement model.

Convergent and discriminant validity assessments were also conducted to evaluate the validity of the EFL vocational school teacher resilience scale. The results are presented in Table 2. For convergent validity, the AVE values ranged from 0.562 to 0.758, and the composite reliability (CR) values fell between 0.834 and 0.940, exceeding the cutoffs of 0.5 and 0.7, respectively, indicating good convergent validity (Hair et al., 2019). In addition, the EFL vocational school teacher resilience scale possessed high discriminant validity since the square root of the AVE for each subscale was larger than its corresponding correlation coefficients (Hair et al., 2019).

**Table 2:** Results of CFA of EFL vocational school teacher resilience scale.

**Factor**	**Item**	**Convergent validity**			**Discriminant validity (** ***r*** **)**		
		***p***	***CR***	***AVE***	**PD**	**EsD**	**CD**
PD	Q01	0.000	0.834	0.562	**0.750**		
	Q02	0.000					
	Q03	0.000					
	Q04	0.000					
EsD	Q08	0.000	0.940	0.758	0.630	**0.871**	
	Q10	0.000					
	Q11	0.000					
	Q12	0.000					
	Q13	0.000					
	Q14	0.000					
CD	Q16	0.000	0.915	0.642	0.585	0.788	**0.801**
	Q17	0.000					
	Q18	0.000					
	Q19	0.000					
	Q20	0.000					

The bold numbers are the square root value of the *AVE*.

PD, professional dimension; EsD, emotional and social dimension; CD, cultural dimension.

### 4.2 The levels of EFL vocational school teacher resilience

We also explored the levels of EFL vocational school teacher resilience, with results presented in Table 3. The EFL vocational school teachers reported a high level of overall resilience (*M* = 5.26, *SD* = 0.65). The result was consistent with prior research reporting a high level of EFL teacher resilience (Liu, 2024b). The EFL vocational school teachers also reported high levels of the cultural dimension (*M* = 5.41, *SD* = 0.71), emotional and social dimension (*M* = 5.20, *SD* = 0.75), and professional dimension (*M* = 5.16, *SD* = 0.76). There was significant difference between cultural dimension and professional dimension, and between cultural dimension and emotional and social dimension. No significant difference was observed between professional dimension and emotional and social dimension.

**Table 3:** Results of descriptive analysis and one-way ANOVA.

**Professional dimension**		**Emotional and social dimension**		**Cultural dimension**		***F*_(2, 528)_**	**Repeated contrasts**
***M***	***SD***	***M***	***SD***	***M***	***SD***		
5.16	0.76	5.20	0.75	5.41	0.71	25.56^***^	professional dimension < cultural dimension emotional and social dimension < cultural dimension

*N* = 265; ^***^p < 0.001.

## 5 Discussion

This paper examined the internal structure and the overall and dimensional profiles of EFL vocational school teacher resilience. The results elucidated the general characteristics of teacher resilience while highlighting the unique attributes of the EFL vocational education ecosystem and its impact on teacher resilience within this specific context.

### 5.1 The structure of EFL vocational school teacher resilience

This study was designed to validate and examine EFL vocational school teacher resilience, employing a modified version of the *Language Teacher Resilience Scale* (Liu et al., 2024, 2025). The empirical findings unveiled a three-dimensional construct for EFL vocational school teacher resilience, encompassing professional, emotional and social, and cultural dimensions.

Regarding the professional dimension, this study exhibited partially consistent with previous studies (Liu et al., 2024; Liu and Chu, 2024; Mansfield et al., 2012; Mansfield and Wosnitza, 2015). Specifically, it involved fostering students' enthusiasm (Q01), facilitating independent learning (Q02), utilizing a multi-faceted approach to engage in activities (Q03), as well as promoting reflection and planning to enhance their teaching (Q04). The similarity was evident in this dimension, which was pivotal in assessing teachers' professional competence in managing teaching-related stress. Unlike those of academically oriented universities and colleges (Warwas and Helm, 2018) and those of high schools focused on student promotion rate (Liu et al., 2024), the professional demands for vocational school teachers focused on cultivating practical and applied skills in EFL learners. Therefore, this dimension concentrated on the EFL vocational school teachers' employment of their professional knowledge and instructional practice as defenses against the challenges arising from the demands of “tri-professionalism” and adversity in EFL vocational school teaching settings. In this study, EFL vocational school teachers engaged in the ongoing enrichment of their professional knowledge through daily teaching and learning activities; they also strived to integrate their English knowledge with the specialized domains-specific knowledge and practice experience. Moreover, EFL vocational school teachers proactively strived to acquire knowledge and insights from student feedback (Özdemir et al., 2024) to strengthen student motivation and engagement in learning English. The proactive professional learning process undertaken by teachers underscored their dedication and optimism to a certain extent. As evidenced in previous research, resilient teachers were often characterized by heightened professional optimism, which could significantly influence their teaching practices (Gu and Li, 2013). Furthermore, teacher practice, an ongoing problem-solving process, represented teacher engagement and adjustment in teaching contexts (Gan et al., 2022). Implementing self-regulated learning promoting strategies within the vocational school has uncovered evidence that it is suitable for meeting students' needs in terms of professional English knowledge and skills (Qiu et al., 2024) and promoting professional self-growth for EFL vocational school teachers.

The emotional and social dimension emerged as an incorporated and unique dimension of EFL vocational school teacher resilience, setting itself apart from the studies of Liu et al. (2024), Mansfield and Wosnitza (2015), and Mansfield et al. (2012). The noteworthy aspect of this dimension lay in its emphasis on the ability to regulate emotion and seek social support to cope with work-related stress, but more importantly, it focused on achieving success (Day and Hong, 2016; Li and Lv, 2022). On the one hand, this dimension accentuated the intrinsic capabilities of EFL vocational school teachers in regulating and managing their emotions (Liu et al., 2024; Mansfield and Wosnitza, 2015), which were critical for maintaining a conducive learning environment and fostering positive educational outcomes (Day and Hong, 2016). In particular, this dimension included the regulation of emotions, the objective discussion of the matter (Q08) and the ability to quickly adapt emotions (Q10). On the other hand, this dimension stated the pivotal abilities of teachers to seek social support in nurturing and bolstering teacher resilience when confronted with adversity and negative emotions (Barnová et al., 2023; Liu et al., 2024; Mansfield and Wosnitza, 2015), including seeking support from colleagues (Q11), integrating into the teaching and researching teams (Q12), actively communicating with others, resolving conflicts (Q13), and balancing work and family life (Q14). The combined dimension outlined the unique settings of EFL vocational school teachers, where the complexity of the teaching and learning environment necessitated a high level of emotional intelligence and social interaction skills. The interplay between emotional regulation and social factors underscored the symbiotic relationship between teachers' internal coping mechanisms and the external social support systems contributing to their resilience (Day and Hong, 2016). Specifically, teaching is a profession that entails emotional work, with emotional involvement stemming from continual interaction and communication with colleagues and students (Day and Hong, 2016; Li and Lv, 2022). Social factors significantly shaped the capacity for emotional control and management among teachers, particularly evident in the quality of their relationships with significant others, such as leaders, colleagues and students, as well as the support they perceived from others (Chen, 2021). For example, vocational schools often establish teams or professional learning communities to collaborate on preparing and designing curricular resources to enhance instructional quality and collaborative development (Warwas and Helm, 2018). However, teachers and their colleagues involved in this process exhibited variations in their educational beliefs, instructional approaches and evaluation methods, which potentially provoked emotional fluctuations if not effectively communicated. Conversely, adequate social support and effective communication from significant others fostered the development of positive emotions and mitigated negative ones, thereby contributing to the emergence of positive psychological factors, such as wellbeing (Day and Hong, 2016). Therefore, resilient vocational school teachers, bolstered by effective emotional regulation and social support, contributed significantly to not only bouncing back from setbacks and achieving emotional and social success but also providing students with the necessary technical expertise and positive learning outcomes.

The cultural dimension corresponded to the new dimension in Liu et al.'s (2024) study, which focused on elucidating how teachers engaged with their educational environment within diverse cultural contexts and responded to the demands and expectations of educational and teaching reforms on EFL vocational school teachers. The critical aspects examined included the cultivation of virtue and character in people (Q16), teaching with their hearts (Q17), fostering students' talents (Q18), serving as a role model for students (Q19), and meeting public expectations and requirements (Q20). The cultural dimension emerged as a fundamental and vital component of EFL vocational school teacher resilience. The measurement encompassed both macro-level and micro-level cultural elements, enabling the examination of cultural factors integral to EFL vocational school teacher resilience and distinctive features. At the macro level, the connotation of cultural dimension aligned with the findings of Liu et al. (2024), which emphasized that this dimension enabled a comprehensive examination of the cultural characteristic of teacher resilience in Chinese backgrounds. The respect and recognition accorded to the teaching profession instilled in EFL vocational school teachers a strong sense of vocation and self-worth. They recognized their responsibilities to meet the social expectations of cultivating “Master Craftsmen” who possessed interdisciplinary proficiency in English language knowledge and professional skills (Qiu et al., 2024). It precisely validated teachers' efforts in response to the demands and expectations outlined in national policy documents. At the micro level, the cultural dimension also hinted at the distinctiveness of vocational school environments and students' characteristics. The issue was particularly evident in teachers' limited domain-specific knowledge, the excessive workload that surpassed the prescribed teaching hours, and the lack of proactive thinking and learning motivation among vocational school students who passively acquired knowledge solely for passing exams. Despite these challenges, EFL vocational school teachers have remained committed to staying updated on pertinent requirements and innovative teaching methods aligned with the unique attributes of vocational schools. For example, the prevailing concept and work-integrated learning model spurred EFL teachers to prioritize students' needs and combine classroom instruction with students' practical skills. Their commitment aimed to educate competitive students, enhance their employment opportunities, and broaden the possibilities for their future. By considering macro and micro cultural factors, this dimension emphasized the importance and the necessity of considering a cultural perspective when examining EFL vocational school teacher resilience.

In adapting this scale, we have thoroughly considered the specific educational environment of EFL vocational teachers and the pressures and challenges they encounter. Data analysis confirmed that the EFL vocational school teacher resilience exhibited a tri-dimensional construct. Compared with previous studies on scale development and validation based on Mansfield and Wosnitza (2015), such as Peixoto et al. (2020), Chen and Lee (2022), and Liu et al. (2024), this study retained the professional dimension (Chen and Lee, 2022; Liu et al., 2024; Mansfield and Wosnitza, 2015; Peixoto et al., 2020) and cultural dimension (Liu et al., 2024), while integrating the emotional and social dimensions into one dimension. These differences manifested in the unique working environments of EFL vocational schools and the distinct characteristics of EFL vocational school teacher resilience. Therefore, this study provided a reliable instrument for measuring EFL vocational school teacher resilience.

### 5.2 The levels of EFL vocational school teacher resilience

This research revealed a notably high level of EFL vocational school teacher resilience, which aligned with the high levels of teacher resilience reported in previous studies (Liu, 2024b), both overall and across its three dimensions: professional, emotional and social, and cultural dimensions. Despite the significant demands and challenges inherent in tri-professionalism, EFL vocational school teachers have exhibited a high level of resilience, which can be attributed to internal and external factors (Barnová et al., 2023; Boldrini et al., 2018; Kärner et al., 2021b; Liu and Chu, 2022a).

Teaching experience ranks prominently among the internal factors contributing to the high EFL vocational school teacher resilience level. Notably, 224 participants, constituting ~84.5% of the total valid sample, possessed over 10 years of teaching experience in this research. The inclusion of such a large subset of veteran teachers offered insight into the understanding of resilience levels. Their extensive experience, encompassing a broad range of accumulated English language and cultural knowledge, occupational pedagogy knowledge, and domain-specific knowledge and practical skills, provided a foundation for addressing professional challenges and equipped them with diverse strategies. These strategies, such as encouraging students with learning difficulties and managing classroom discipline, enabled them to skillfully handle and navigate the inevitable stresses and pressures in the classroom environment over time, enhancing their resilience in the face of teaching-related difficulties. Additionally, experienced EFL vocational school teachers have developed extensive interpersonal connections within and beyond schools. By engaging in active communication and collaboration with important others within the school environment, these teachers effectively mitigated negative emotions and teaching- and researching-related tension. Beyond the school setting, experienced teachers have established close partnerships with industry professionals, allowing them to consult experts for practical skill-related challenges and setbacks. Furthermore, experienced teachers were characterized by a deep sense of professional identity, job satisfaction and vocation, which served as an internal catalyst when facing challenges (Boldrini et al., 2018; Liu et al., 2024). They fully recognized their responsibility to enhance students' English proficiency and cross-cultural communication skills while cultivating technical talent. This stimulated teachers to sustain their passion and motivation while facilitating timely reflection and adjustment of instructional approaches, thereby maintaining a high level of resilience. In a nutshell, the accumulation of teaching experience in vocational schools emerged as a prominent factor affecting the resilience level, consistent with previous research suggesting that teachers with more years of work experience exhibited higher levels of resilience than novice teachers (Barnová et al., 2023).

Additionally, positive psychological factors have been consistently recognized to influence teacher resilience levels significantly (Duan et al., 2023; Mansfield et al., 2016), including agency, self-efficacy, hope, etc. (Oxford, 2016). Teacher self-efficacy, for example, was regarded as “a teacher's judgment of his or her competence in managing the classroom, engaging students and performing assigned teaching tasks” (Liu, 2024a, p. 74). Teacher self-efficacy is not only a crucial component of EFL teacher resilience (Li et al., 2019) but also a significant internal influential factor and predictor of EFL teacher resilience (Wang et al., 2024; Wang and Pan, 2023). Specifically, EFL vocational school teachers with higher self-efficacy exhibit confidence in their abilities to bounce back from challenges by augmenting their job satisfaction and facilitating the adoption of effective instructional strategies. They believe they can effectively handle tri-professionalism demands and classroom challenges, better mitigate negative emotions stemming from students' misbehavior and inadequate support and positively engage with others within the complex ecosystem of vocational foreign language education. For instance, the insufficient attention to English learning by vocational school students and their prior negative learning experiences have led to a lack of motivation for English learning and misbehavior in English classrooms. These issues generate negative emotions among EFL vocational school teachers. However, teachers with higher self-efficacy are more likely to believe they can overcome these challenges by applying various strategies, such as adapting teaching methods, building positive teacher-student relationships, and collaborating with colleagues. The confidence reflects a higher level of resilience when addressing pressure and challenges.

The influence of external environmental factors on the level of EFL vocational school teacher resilience is also pertinent (Boldrini et al., 2018; Kärner et al., 2021b). Regarding cultural factors, cooperation is regarded as a fundamental social behavior and value orientation within traditional Chinese values. Despite resource limitations, EFL vocational school teachers in China proactively leverage their initiative to seek or provide social support from or for important others through communication and cooperation, fostering a cooperative working environment. Previous research has also confirmed the vital role of social interaction in enhancing teacher resilience (Liu et al., 2024; Liu and Chu, 2022a). Additionally, although prior studies have highlighted the issue of low recognition for vocational school teachers (Boldrini et al., 2018; Chen et al., 2023), with the growing significance of vocational education within China's higher education system (Wen and Zhang, 2021) and its positive impact on promoting educational equity (Ye et al., 2023), social cognition and attitudes toward vocational education have progressively shifted in a positive direction. Consequently, the status of vocational school teachers has been elevated. The growing social recognition of EFL vocational school teachers not only enhances their sense of achievement but also significantly contributes to their ability to manage pressure and challenges, thereby ensuring high-quality retention and exemplifying high resilience characteristics.

Regarding policy factors, the Chinese government has enacted a comprehensive set of policies that underscore the importance of vocational schools and outline the developmental trajectory for the English curriculum at the vocational schools (Wen and Zhang, 2021), as well as facilitate teachers' professional growth and sustainable development (Chen et al., 2023). These policies provide policy guarantees for enhancing the level of EFL vocational school teacher resilience. To facilitate the effective implementation of these policies, vocational schools have organized training programs designed to continuously improve teachers' professional competencies. Although the current training initiatives may not fully meet all teachers' needs, they have fostered a sense of support among teachers, increasing their confidence in addressing pressures and challenges. To sum up, cultural and policy factors collectively impact the level of EFL vocational school teacher resilience, contributing to a positive school environment and a supportive vocational education ecosystem. That, in turn, enables teachers to more effectively address professional challenges and achieve the concurrent achievement of personal and collective growth.

## 6 Conclusions and implications

This study examined the internal structure and level of EFL vocational school teacher resilience employing the scale adapted by Liu et al. (2024, 2025), echoing the multidimensional framework of teacher resilience (Chen, 2024; Liu et al., 2024; Liu and Chu, 2022a, 2024; Mansfield et al., 2012; Mansfield and Wosnitza, 2015). The results indicated that EFL vocational school teacher resilience was a tri-dimensional construct involving professional, emotional and social, and cultural dimensions. In the context of vocational school English teaching, teacher resilience was defined as the quality to confront teaching- and researching-related challenges in vocational schools effectively. In addition, it was a dynamic process that dynamically interacted with the various contextual factors in their teaching and living environment to promote their sustainable professional development. It was obviously demonstrated through the agency to expand professional knowledge, meeting the requirements of tri-professionalism (professional dimension), utilizing strategies for emotional regulation to actively communicate with significant others and gain their support (emotional and social dimension), and actively responding to social expectations and requirements (cultural dimension). Moreover, the high level of resilience demonstrated by EFL vocational school teachers overall and across all dimensions reflected solid subject knowledge, proficient emotional management skills, and a strong sense of responsibility among EFL vocational school teachers.

Considering the context-specific nature of teacher resilience (Gao et al., 2022; Gu and Li, 2013; Liu et al., 2024) and the unique working environment of EFL vocational school teachers, we adapted and validated a scale specifically for EFL vocational school teacher resilience. This scale's reliability and validity were rigorously tested through quantitative data analysis, providing a robust and theoretically grounded measurement tool for EFL teachers in vocational schools. This research not only theoretically enriched the understanding of teacher resilience at specific educational stages and disciplines but also offered practical insights and evidence-based recommendations to enhance EFL vocational school teacher resilience.

These findings from this study have significant implications for the professional development of EFL vocational school teachers, which can be extracted into three pivotal recommendations. First, it is imperative for EFL vocational school teachers to prioritize enhancing their competencies to meet the demands of tri-professionalism; they should utilize their effective communication skills to foster collaboration with non-EFL subject teachers and gain a comprehensive understanding of students' English learning needs. Second, vocational schools are encouraged to cultivate a strong sense of awareness among students regarding the value of English courses within the vocational curriculum. Concurrently, specialized training programs should be instituted for English teachers, enabling them to deepen their expertise in their respective teaching fields. Schools can also introduce targeted incentives to motivate teachers to pursue “dual-professional teacher” roles, enriching their professional profiles. Finally, it is necessary to encourage policymakers to direct greater attention toward English courses in vocational schools and equip teachers with pedagogical resources and textbooks that are more closely aligned with the practical demands of their specific professional fields addressing the practical challenges teachers encounter. Furthermore, establishing professional learning communities has been seen as an effective method to improve vocational school teachers' professional learning (Warwas and Helm, 2018).

However, this study has limitations. First, the present study utilized a quantitative approach without assessing criterion validity. Future research could include additional measures to establish the validity and incorporate qualitative methods to provide a more nuanced understanding of EFL vocational school teacher resilience. Second, it is essential to address the limitations of relatively small sample size, imbalanced gender ratios and incomplete coverage of regions to enhance the representativeness of the teaching population and the universal applicability and reliability of the scale. Finally, future research should explore how EFL vocational school teacher resilience correlates with other psychological factors. This exploration would deepen the understanding of the influences on teacher resilience and its role in other psychological aspects, enhancing research on EFL vocational school teacher psychology.

## Data Availability

The data analyzed in this study is subject to the following licenses/restrictions: Further inquiries can be directed to the first author. Requests to access these datasets should be directed to siyuduan@nenu.edu.cn.
