# Supplementary material for: Examining EFL vocational school teacher resilience in the Chinese context: a structural equation modeling approach
Source: Front Psychol. 2025 Nov 19;16:1444979. doi: 10.3389/fpsyg.2025.1444979 (PMC12673808; doi:10.3389/fpsyg.2025.1444979)
Supplement: Supplementary file 1 [file Data_Sheet_1.docx]

**APPENDIX**

**Appendix for Results of univariate normality test.**

| Items | Skewness | | Kurtosis | |
| --- | --- | --- | --- | --- |
|  | *M* | SE | *M* | SE |
| Q01 | −1.467 | 0.150 | 3.155 | 0.298 |
| Q02 | −1.367 | 0.150 | 2.856 | 0.298 |
| Q03 | −1.221 | 0.150 | 1.808 | 0.298 |
| Q04 | −0.878 | 0.150 | 0.287 | 0.298 |
| Q05 | −1.260 | 0.150 | 1.708 | 0.298 |
| Q06 | −0.928 | 0.150 | 0.900 | 0.298 |
| Q07 | −0.968 | 0.150 | 1.103 | 0.298 |
| Q08 | −1.353 | 0.150 | 2.328 | 0.298 |
| Q09 | −1.203 | 0.150 | 1.248 | 0.298 |
| Q10 | −1.372 | 0.150 | 2.838 | 0.298 |
| Q11 | −0.953 | 0.150 | 1.054 | 0.298 |
| Q12 | −1.174 | 0.150 | 1.480 | 0.298 |
| Q13 | −1.098 | 0.150 | 1.722 | 0.298 |
| Q14 | −1.146 | 0.150 | 1.781 | 0.298 |
| Q15 | −0.977 | 0.150 | 0.706 | 0.298 |
| Q16 | −1.152 | 0.150 | 0.989 | 0.298 |
| Q17 | −1.723 | 0.150 | 3.834 | 0.298 |
| Q18 | −1.621 | 0.150 | 3.518 | 0.298 |
| Q19 | −1.481 | 0.150 | 3.065 | 0.298 |
| Q20 | −0.757 | 0.150 | −0.558 | 0.298 |

Note. *N* = 265.

**Appendix for Results of independent samples t−test**

| Items | Bottom 27%  (*n* = 78) | | Top 27%  (*n* = 76) | | *MD* | *t* (152) |
| --- | --- | --- | --- | --- | --- | --- |
|  | *M* | *SD* | *M* | *SD* |  |  |
| Q01 | 4.55 | 0.99 | 5.84 | 0.46 | −1.29 | −10.33^***^ |
| Q02 | 4.46 | 1.05 | 5.92 | 0.32 | −1.46 | −11.58^***^ |
| Q03 | 4.14 | 1.15 | 5.82 | 0.45 | −1.68 | −11.85^***^ |
| Q04 | 4.49 | 0.85 | 5.95 | 0.23 | −1.46 | −14.51^***^ |
| Q05 | 4.03 | 1.28 | 5.78 | 0.53 | −1.75 | −11.04^***^ |
| Q06 | 4.06 | 1.00 | 5.82 | 0.42 | −1.75 | −14.12^***^ |
| Q07 | 3.88 | 0.98 | 5.91 | 0.29 | −2.03 | −17.27^***^ |
| Q08 | 4.59 | 0.93 | 5.95 | 0.28 | −1.36 | −12.18^***^ |
| Q09 | 4.26 | 0.97 | 5.91 | 0.41 | −1.65 | −13.68^***^ |
| Q10 | 4.29 | 0.96 | 5.95 | 0.23 | −1.65 | −14.69^***^ |
| Q11 | 4.13 | 0.87 | 5.93 | 0.25 | −1.81 | −17.35^***^ |
| Q12 | 4.23 | 0.93 | 5.99 | 0.12 | −1.76 | −16.43^***^ |
| Q13 | 4.50 | 0.86 | 5.96 | 0.20 | −1.47 | −14.51^***^ |
| Q14 | 4.46 | 0.91 | 5.88 | 0.40 | −1.42 | −12.52^***^ |
| Q15 | 4.54 | 0.77 | 5.99 | 0.12 | −1.45 | −16.27^***^ |
| Q16 | 4.71 | 0.74 | 6.00 | 0.00 | −1.30 | −15.24^***^ |
| Q17 | 4.67 | 0.96 | 5.96 | 0.20 | −1.29 | −11.49^***^ |
| Q18 | 4.64 | 0.90 | 6.00 | 0.00 | −1.36 | −13.21^***^ |
| Q19 | 4.62 | 0.87 | 5.99 | 0.12 | −1.37 | −13.61^***^ |
| Q20 | 4.55 | 0.71 | 5.99 | 0.12 | −1.44 | −17.30^***^ |

*Note.* ^***^*p* < 0.001.

**Appendix for Results of item−total correlation analysis**

| Items | The overall scale |
| --- | --- |
|  |  |
| Q01 | 0.594^**^ |
| Q02 | 0.656^**^ |
| Q03 | 0.615^**^ |
| Q04 | 0.702^**^ |
| Q05 | 0.613^**^ |
| Q06 | 0.694^**^ |
| Q07 | 0.764^**^ |
| Q08 | 0.714^**^ |
| Q09 | 0.712^**^ |
| Q10 | 0.781^**^ |
| Q11 | 0.833^**^ |
| Q12 | 0.822^**^ |
| Q13 | 0.771^**^ |
| Q14 | 0.704^**^ |
| Q15 | 0.766^**^ |
| Q16 | 0.755^**^ |
| Q17 | 0.718^**^ |
| Q18 | 0.797^**^ |
| Q19 | 0.799^**^ |
| Q20 | 0.824^**^ |

*Note.* ^**^*p* < 0.01.

**Appendix for items in EFL Vocational School Teacher Resilience Scale**

Q01 When students lack interest in English learning, I use my professional knowledge to adjust teaching methods to arouse their enthusiasm.

Q02 When faced with teaching/research difficulties, I improve my professional competence and practical skill through independent learning to overcome difficulties.

Q03 When faced with the bottleneck period at professional development, I actively participate in varieties activities (such as domain−specific training programs, academic conferences and practical activities) to seek breakthroughs.

Q04 When faced with shortcomings in work, I reflect on them, make plans and gradually improve them.

Q05 When faced with stress caused by promotion, I work hard in teaching/research work to meet the requirement.

Q06 When faced with frustrating or irritating events at school, I can stay calm.

Q07 When encountering difficulties in teaching/research, I actively deal with them, because I firmly believe that adversity helps people grow.

Q08 When students make mistakes, I can control my emotions and talk about it.

Q09 In the English class, I do not easily show my negative emotions because of students’ misbehavior.

Q10 When encountering sad or irritating events, I can adjust my emotions in a short time without affecting my teaching.

Q11 When encountering difficulties in English teaching/research, I seek the help of my colleagues and actively cope with the challenges.

Q12 I can integrate well into the English teaching/research team, overcome work difficulties, and seek personal development.

Q13 When encountering disagreements with others, I can effectively resolve the conflict by seeking help from others.

Q14 When encountering the work−life imbalance, I can communicate well with my family and balance my work and life.

Q15 When teaching is not working well, I communicate with students and improve the teaching based on their feedback.

Q16 The educational mission of establishing virtue and cultivating people enables me to teach and work hard even when I encounter difficulties.

Q17 Teaching is the most glorious profession under the sun, even if the salary is limited, I teach with my heart.

Q18 It is the responsibility of teachers to cultivate students to become talents possessing interdisciplinary proficiency in English language knowledge and professional skills, so I can go forward and do my job well no matter how many challenges I encounter.

Q19 A teacher should be a model, so no matter what difficulties arise in teaching, I try to play an exemplary role and set an example for my students.

Q20 In the face of the increasing expectations and requirements imposed by the reform of English education in vocational schools, I can work hard and fulfil my mission.

Note: This study built upon the questionnaire developed by Liu et al. (2024) and adapted it to the context of EFL vocational school education. Specifically, we revised their original questionnaire to create a 20−item instrument tailored to the unique characteristics of EFL vocational school teacher resilience. The revised questionnaire initially comprised four dimensions, namely professional dimension (Q01−Q05); emotional dimension (Q06−Q10); social dimension (Q11−Q15); cultural dimension (Q16−Q20).

After conducting Confirmatory Factor Analysis, the final scale retained 15 items and three dimensions, which were as follows: professional dimension (Q01−Q04); emotional and social dimension (Q08, Q10−Q14); cultural dimension (Q16−Q20).
